# Ingested Foreign Body Causing Duodenal and Colonic Perforations in a Child

**DOI:** 10.21699/ajcr.v8i3.546

**Published:** 2017-05-01

**Authors:** Dileep Garg, Aditya Pratap Singh, Sunil Kothari

**Affiliations:** 1 Department of Pediatric Surgery, SN Medical College Jodhpur, Rajasthan, India; 2 Department of Pediatric Surgery, SMS Medical College Jaipur, Rajasthan, India

**Keywords:** Duodenum, Hair clip, Foreign body, Perforation

## Abstract

Most ingested foreign bodies usually pass uneventfully through the gastrointestinal tract. Few may cause complications and require surgical interventions. We report a 1.5-year-old child who ingested hair clip and presented with vomiting and obstruction. At operation, we found duodenal and colonic perforations. Hair clip was removed with repair of perforations.

## CASE REPORT

An 18-month-old female infant admitted with complaints of abdominal pain, constipation, and bilious vomiting for two days. On examination, abdomen was distended and tender. Abdominal x-rays revealed dilated bowel loops and a hair clip in the abdominal cavity (Fig.1). Laboratory investigations were within normal ranges. Ultrasound abdomen revealed dilated bowel loops. On exploration, there was bilious fluid was present in peritoneal cavity. Hair clip was noted in fourth part of duodenum. Two ends of hair clip were protruding through two duodenal perforations (1 cm apart). On further exploration, a perforation of sigmoid colon was also present. Duodenum was mobilized and hair clip was removed (Fig.1). Duodenum was closed in single layer with interrupted sutures. Colonic perforation was also repaired. Peritoneal lavage was done with normal saline and abdomen closed in layers after drain placement. Postoperative course was uneventful.

**Figure:**
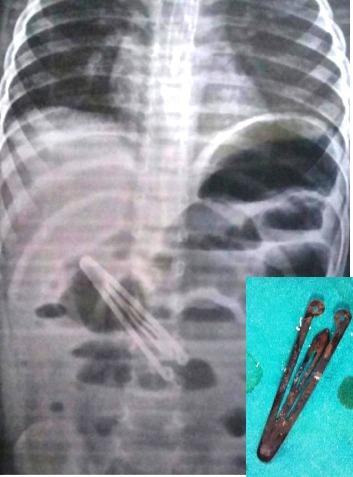
Figure 1: Hair clip seen in x-ray abdomen. Inset shows retrieved hair clip.

## DISCUSSION

Foreign body (FB) ingestion is a common clinical problem in young children. Commonly ingested objects are coins, jewelry, button-type batteries, needles and pins [1]. FB ingestion may cause complications in less than 1% of cases [1]. Perforation of the gastrointestinal tract may lead to peritonitis [2]. Duodenal perforation in children due to ingested toothbrush and lollipop stick has been reported [3,4]. In our case, the hair clip was entrapped in fourth part of duodenum and resulted in perforation with protrusion of two sharp prongs of clip that lead to colonic perforation as well. To conclude, ingested hair clip in children may cause life threatening complications. Such objects should be kept away from kids.

## Footnotes

**Source of Support:** Nil

**Conflict of Interest:** None declared

